# Tobacco Use, Exposure to Secondhand Smoke, and Cessation Counseling Among Health Professions Students: Greek Data from the Global Health Professions Student Survey (GHPSS).

**DOI:** 10.3390/ijerph9010331

**Published:** 2012-01-19

**Authors:** Anastasia Barbouni, Christos Hadjichristodoulou, Kyriakoula Merakou, Eleni Antoniadou, Kallirrhoe Kourea, Evangelia Miloni, Charles W. Warren, George Rahiotis, Jenny Kremastinou

**Affiliations:** 1 Department of Public Health, National School of Public Health, Alexandras Av. 196, 11521, Athens, Greece; Email: kmerakou@esdy.edu.gr (K.M.); eantoniadou@gmail.com (E.A.); publichealth@esdy.edu.gr (K.K.); m-kelly@live.com (E.M.); jkremastinou@esdy.edu.gr (J.K.); 2 Department of Hygiene and Epidemiology, Faculty of Medicine, University of Thessaly, Papakyriazi 22, 41222, Larissa, Greece; Email: xhatzi@med.uth.gr (C.H.); grach@med.uth.gr (G.R.); 3 Office of Smoking and Health, National Center for Chronic Disease Prevention and Health Promotion, Centers for Disease Control and Prevention, 1600 Clifton Rd. Atlanta, GA 30333, USA; Email: wcw1@cdc.gov

**Keywords:** tobacco, smoking, prevalence, students, secondhand smoke, health profession, cessation, survey

## Abstract

We conducted the GHPSS (Global Health Professions Student Survey) to obtain information regarding health profession students’ smoking habits and perceptions, exposure to secondhand smoke (SHS) as well as level of knowledge and training on tobacco use and smoking cessation counseling. GHPSS is a survey for third-year students in the following fields: health visitors, dentistry, medicine, nursing and/or pharmacy. The highest tobacco use prevalence rate and exposure to SHS were recorded among health visitor students with 46.4% and 33.3% respectively. The majority of the respondents believed that their profession serves as a role model for their patients. Formal training on cessation counseling ranged between 10.7% for health visitor students to 22.4% for nursing students. The relatively high percentage of health profession students who currently smoke and the alarmingly high percentage of those exposed to SHS indicate lack of concerted efforts for implementation and effective enforcement of the anti-tobacco policy measures. Despite its significance, formal training on cessation counseling for students is strikingly low. These results indicate the urgent need to train health professional students on tobacco cessation counseling and educate them on the dangers of tobacco use, SHS and the positively influential role they can play to affect their patients’ smoking habits.

## 1. Introduction

Tobacco use is one of the major preventable causes of premature death and disease in the World. A disproportionate share of the global tobacco burden falls on developing countries, where 84% of the current 1.3 billion smokers reside [1]. The World Health Organization (WHO) attributes approximately 5 million deaths a year to tobacco. The number is expected to exceed 8 million deaths by 2030, with approximately 70% of these deaths occurring in developing countries [2]. In Greece, smoking prevalence among adults is the highest in Europe, with 42% of current smokers and tobacco consumption is the second highest in the region, with 21.4 cigarettes per smoker per day [3]. The above findings and projections are particularly worrying and health professionals can play a critical role in tobacco control by providing effective interventions against tobacco use by counseling their patients to quit smoking. 

Health professions students have been found to play an important role in cessation and prevention of tobacco use among their patients [4,5,6,7]. Counseling by health professions students has been shown to increase smoking cessation [4]. Despite the involvement of health professions students, as the largest group of healthcare professionals in tobacco control, only a few studies have collected information on tobacco use, exposure to secondhand smoke, and training to provide cessation counseling among health professions students. These studies used different sampling methods, questionnaires, and data collection procedures, and very few are from low or middle-income countries [8,9,10,11]. The WHO, US Centers for Disease Control and Prevention, and the Canadian Public Health Association have attempted to overcome these limitations by developing and implementing the Global Health Professions Student Survey (GHPSS) [12]. The GHPSS includes surveys of dental, medical, nursing, pharmacy students and other health professions deemed appropriate by each country.

The following report discusses data from the GHPSS conducted among 3rd year medical, dental, pharmacy, nursing, health visitor and nutrition students in Greece. 

The main objectives of the study were:

1. To measure tobacco use and exposure to SHS among health professions students in Greece
2. To estimate health professions students’ beliefs towards tobacco, their undergraduate education on tobacco control and their perception of their role in patient smoking cessation

## 2. Methods

### 2.1. Design

The GHPSS is part of the Global Tobacco Surveillance System, which collects data through four surveys: the Global Youth Tobacco Survey, the Global School Personnel Survey, the Global Adult Tobacco Survey, and the GHPSS. The GHPSS is a school-based survey of 3rd year students pursuing advanced degrees in dentistry, medicine, pharmacy, nursing and other health professions. The GHPSS uses a core questionnaire on demographics, prevalence of cigarette smoking and use of other tobacco products, exposure to secondhand smoke (SHS), desire to quit smoking, and training received to provide patient counseling on cessation techniques. The GHPSS has a standardized methodology for selecting participating schools and uniform data processing procedures [12]. 

All schools that offer a degree in medicine, dentistry, pharmacy, nursing, health visiting and nutrition were eligible to participate in the survey. In Greece, seven schools offer medical degrees; two schools offer dental degrees, two pharmacy degrees and six nursing degrees. There are also two separate schools for nutrition and health visitors. Overall, fourteen out of nineteen schools agreed to participate in the GHPSS study. Ten of those (with a total of 854 students responding) participated during the period October 2009 to May 2010 and the remaining four during the following academic year (October 2010 to May 2011). The Greek GHPSS was conducted in schools during regular lectures and class sessions. Anonymous, self-administered data collection procedures were used. The final questionnaire was translated into Greek and back-translated into English to check for accuracy. The questionnaires were gathered and sent to CDC for statistical analysis.

This research study was conducted in compliance with the Helsinki Declaration and approval was granted by the Bioethics Committee of the National School of Public Health, Athens, Greece.

### 2.2. Measurement

This report includes information on current cigarette smoking, current use of tobacco products, exposure to SHS at home and in public places, and the extent to which schools have official policies banning smoking in school buildings and clinics, and if the policies are enforced. In addition, attitude questions were asked regarding: health professionals as role models for their patients, whether health professionals think they should get training in patient cessation techniques, and if they have ever received formal training on such cessation counseling techniques. 

## 3. Results

### 3.1. Schools’ Overall Response Rates

The school response rates for the Greek GHPSS were 42.9% for medical schools, 50.0% for dental schools, 50.0% for pharmacy schools, and 100% for nursing, health visitor and nutrition schools (Table 1). The student response rate for the Greek GHPSS was 89.3% for medical students, 94.2% for dental students, 54.5% for pharmacy students, 78.5% for nursing students, 52.8% for health visitor students and 100% for nutrition students. 

**Table 1:** Response Rates of Colleges and Third-Year Medical, Dental, Pharmacy, Nursing, Health Visitor and Nutrition Students (Greek Ghpss, 2009).

	Medical	Dental	Pharmacy	Nursing	Health Visitor	Nutrition
Schools (%)	42.9%	50.0%	50.0%	100.0%	100.0%	100.0%
Schools (*n*)	3/7	1/2	1/2	3/3	1/1	1/1
Students (%)	89.3%	94.2%	54.5%	78.5%	52.8%	100%
Students (*n*)	325/364	113/120	60/110	226/288	28/53	101/101

### 3.2. Student Characteristics

The percentage of medical students who were females was 57.6% and 92.5% were between 19 and 24 years of age, while 62.8% of dental students were females and 90.2% were between 19 and 24 years of age. The percentage of pharmacy students who were females was 76.3% and 96.6% were between 19 and 24 years of age, but 84.5% of nursing students were females and 90.6% were between 19 and 24 years of age. Health visitor students who were females was 71.4% and 82.1% of them were between 19 and 24 years of age. Finally, the percentage of nutrition students who were females was 82.0% and 92% were between 19 and 24 years of age. 

### 3.3. Tobacco Use

Among pharmacy students, 27.1% currently smoked cigarettes while health visitor students peaked at 46.4% (Table 2). The prevalence for current cigarette smoking among medical students is 28.8%, 39.1% for dental students, 30.9% for nursing students and 33.3% for nutrition students. 

Among pharmacy students, less than 28.8% currently used tobacco products while the percentage of health visitor students demonstrated the highest prevalence with 46.4% (Table 2). The prevalence for tobacco use among medical students is 29.5%, 38.7% for dental students, 31.8% for nursing students and 35.0% for nutrition students.

**Table 2:** Lifetime and Current Prevalence of Tobacco Use among Third-Year Health Professional Students (Greek Ghpss, 2009).

	All Respondents				Current Use	
	Ever smoked cigarettes	Age of first cigarette		Ever used tobacco products	Cigarettes	Any tobacco products
		11–15 years	18–19 years			
	% (95% CI)	% (95% CI)		% (95% CI)	% (95% CI)	% (95% CI)
**Medical Students**						
Total	54.4 (49.1–59.6)	7.1 (4.8–10.4)	23.0 (18.8–27.8)	57.2 (51.9–62.4)	28.8 (24.2–33.8)	29.5 (24.9–34.6)
Women	48.2 (41.1–55.3)	4.1 (1.7–7.6)	21.6 (16.2–28.0)	49.5 (42.4–56.5)	25.9 (19.8–32.3)	25.7 (19.7–32.1)
Men	63.2 (54.8–70.7)	11.3 (6.8–17.5)	24.7 (18.0–32.4)	68.1 (60.1–75.5)	32.7 (25.4–41.0)	34.6 (27.1–42.9)
**Dental Students**						
Total	74.3 (65.4–81.8)	9.9 (5.4–17.1)	26.1 (18.6–35.1)	77.0 (68.2–84.0)	39.1 (30.4–48.7)	38.7 (30.1–48.3)
Women	71.8 (60.5–81.8)	8.6 (3.0–16.8)	27.1 (17.3–38.5)	73.2 (62.0–82.9)	43.5 (32.0–55.7)	42.9 (31.5–55.0)
Men	78.6 (62.9–88.8)	12.2 (3.9–25.0)	24.4 (13.2–40.4)	83.3 (70.0–93.4)	31.7 (18.7–47.8)	31.7 (18.7–47.8)
**Pharmacy Students**						
Total	55.9 (45.9–65.3)	8.5 (3.9–15.2)	28.8 (20.4–38.2)	64.4 (54.9–73.1)	27.1 (18.8–36.3)	28.8 (20.4–38.2)
Women	53.3 (42.3–64.7)	8.9 (3.2–16.8)	26.7 (17.5–37.5)	62.2 (51.0–72.8)	24.4 (15.5–35.0)	24.4 (15.5–35.0)
Men	64.3 (43.4–82.4)	7.1 (1.0–25.3)	35.7 (17.6–56.6)	71.4 (51.2–88.1)	35.7 (17.6–56.6)	42.9 (23.7–63.7)
**Nursing Students**						
Total	62.2 (56.3–67.9)	11.6 (8.2–16.0)	19.2 (14.7–24.3)	62.3 (56.4–68.0)	30.9 (25.5–36.6)	31.8 (26.5–37.7)
Women	62.0 (55.6–68.2)	13.7 (9.7–18.9)	19.4 (14.5–25.0)	62.0 (55.6–68.2)	30.6 (24.8–37.0)	30.7 (24.9–37.0)
Men	62.2 (45.7–76.5)	0.0 (0.0–8.2)	18.8 (8.4–33.5)	63.1 (46.8–77.1)	30.5 (17.3–46.3)	35.7 (21.2–51.4)
**Health Visitor Students**						
Total	64.3 (49.9–76.9)	7.1 (2.1–18.1)	25.0 (13.8–38.4)	64.3 (49.9–76.9)	46.4 (33.0–61.0)	46.4 (33.0–61.0)
Women	60.0 (43.0–75.8)	10.0 (2.9–24.8)	20.0 (9.5–37.1)	60.0 (43.0–75.8)	35.0 (19.9–51.8)	35.0 (19.9–51.8)
Men	75.0 (45.9–92.4)	0.0 (0.0–21.6)	37.5 (15.8–66.2)	75.0 (45.9–92.4)	75.0 (45.9–92.4)	75.0 (45.9–92.4)
**Nutrition Students**						
Total	63.9 (53.5–73.4)	7.1 (2.9–14.2)	17.3 (10.4–26.3)	65.0 (54.8–74.3)	33.3 (24.2–43.5)	35.0 (25.7–45.2)
Women	62.5 (51.0–73.1)	8.8 (3.6–17.2)	15.0 (8.0–24.7)	64.6 (53.3–74.9)	30.9 (21.1–42.1)	32.9 (22.9–44.2)
Men	70.6 (44.0–89.7)	0.0 (0.0–18.5)	27.8 (9.7–53.5)	66.7 (41.0–86.7)	44.4 (21.5–69.2)	44.4 (21.5–69.2)

### 3.4. Exposure to Secondhand Smoke (SHS)

Among medical students, 21.5% reported that they had been exposed to SHS in their home on each of the past seven days. Similarly, 20.9% of dental students, 16.3% among pharmacy students, 23.7% among nursing students, 33.3% of health visitor students and 28.8% of nutrition students reported SHS exposure at home on each of the past seven days (Table 3).

Among health visitor students, 53.3% reported that they had been exposed to SHS in public places on each of the past seven days. Similarly, 46.3% of dental, 4.2% among pharmacy students, 39.6% among medical students, 30.3% among nutrition students and 28.3% among nursing students reported being exposed to SHS in public places on each of the past seven days (Table 3).

**Table 3:** Exposure to Secondhand Smoke (only for non-current smokers) among Third-Year Health Professional Students.

Exposure to smoke at home during the past seven days					
	0 days	1–2 days	3–4 days	5–6 days	all 7 days
	% (95%CI)	% (95%CI)	% (95%CI)	% (95%CI)	% (95%CI)
Medical	31.7 (26.0–37.8)	20.6 (15.8–26.1)	19.3 (14.7–24.8)	7.0 (4.3–11.0)	21.5 (16.5–27.0)
Dental	29.9 (19.4–41.6)	7.5 (2.3–15.7)	23.9 (14.6–35.5)	17.9 (10.1–29.1)	20.9 (12.3–32.3)
Pharmacy	37.2 (26.3–48.6)	16.3 (9.1–26.5)	14.0 (7.2–23.6)	16.3 (9.1–26.5)	16.3 (9.1–26.5)
Nursing	32.4 (25.7–39.3)	18.8 (13.4–24.9)	19.5 (14.3–26.0)	5.6 (2.9–10.0)	23.7 (18.0–30.4)
Health Visitor	20.0 (8.1–40.1)	20.0 (8.1–40.1)	20.0 (8.1–40.1)	6.7 (0.9–23.1)	33.3 (15.9–52.5)
Nutrition	33.3 (22.2–46.0)	9.1 (3.4–18.7)	18.2 (9.8–29.6)	10.6 (4.4–20.6)	28.8 (18.3–41.3)
**Exposure to smoke in public places during the past seven days**					
Medical	5.2 (2.8–8.7)	21.0 (16.1–26.5)	24.2 (19.0–29.9)	10.0 (6.5–14.3)	39.6 (33.5–45.9)
Dental	6.0 (1.6–13.8)	10.4 (4.1–19.3)	19.4 (11.2–30.7)	17.9 (10.1–29.1)	46.3 (34.4–58.5)
Pharmacy	0.0 (0.0–4.6)	18.6 (11.0–29.3)	27.9 (18.4–39.1)	9.3 (3.7–17.5)	44.2 (33.1–55.9)
Nursing	13.3 (9.0–19.1)	25.2 (19.4–32.1)	19.5 (14.3–26.0)	13.6 (9.0–19.1)	28.3 (22.2–35.4)
Health Visitor	6.7 (0.9–23.1)	20.0 (8.1–40.1)	6.7 (0.9–23.1)	13.3 (3.9–32.1)	53.3 (34.1–72.6)
Nutrition	13.6 (6.4–24.3)	22.7 (13.3–34.7)	18.2 (9.8–29.6)	15.2 (7.5–26.1)	30.3 (19.6–42.9)

The proportion of medical students reporting that their schools have smoking regulations in both school buildings and clinics was 24.2%, 52.2% of dental students, 33.4% of nursing students, 25.0% of health visitor students and 2.0% of nutrition students. Worth noting, however, is the percentage of students responding “I don’t know” regarding smoking regulations. The percentages ranged between 6.8% for pharmacy students to 42.9% for nutrition students. Furthermore, the percentage of students reporting that such policies are enforced ranged from 7.1% among health visitor students to 58.5% among dental students (Table 4).

**Table 4:** Smoking regulations among Third-Year Health Professional Students.

	Schools with smoking regulations in school buildings and clinics			Schools that had an official policy banning smoking in school buildings and clinics that enforced the ban
	Both school buildings and clinics		“I don’t know”	
	% (95%CI)			% (95%CI)
Medical	24.2 (19.9–29.1)	40.4 (35.3–45.7)		15.8 (12.2–20.1)
Dental	52.2 (43.0–61.6)	23.0 (16.0–31.8)		58.5 (48.7–67.6)
Pharmacy	0.0 (0.0–3.4)	6.8 (2.6–12.9)		25.4 (17.2–34.4)
Nursing	33.4 (27.9–39.2)	22.9 (18.2–28.4)		13.7 (10.0–18.4)
Health Visitor	25.0 (13.8–38.4)	35.7 (23.1–50.1)		7.1 (2.1–18.1)
Nutrition	2.0 (0.2–7.2)	42.9 (32.9–53.3)		17.5 (10.6–26.6)
Total	25.4 (22.8–28.2)	29.9 (27.2–32.9)		20.7 (18.3–23.4)

### 3.5. Health Professional Roles and Training

The percentage of medical students who believe health professionals serve as a role model for their patients and the public was 55.4%, 57.1% for dental students, 37.3% for pharmacy students, 70.3% for nursing students, 67.9% for health visitor students and 68.0% for nutrition students (Table 5). The majority of students, ranging between 94.0% among nutrition students and 98.3% among pharmacy students, thought health professionals should receive specialised training on cessation techniques. The percentage of health professions students reporting that they had ever received some kind of formal training in their professional school on cessation approaches to use with their patients ranged from 10.7% among health visitor students to 25.0% among nutrition students. 

**Table 5:** Curriculum/Training among Third-Year Health Professional Students.

Questions	Medical	Dental	Pharmacy	Nursing	Health Visitor	Nutrition
	% (95%CI)	% (95%CI)	% (95%CI)	% (95%CI)	% (95%CI)	% (95%CI)
Do health professionals serve as role models for their patients and the public?	55.4 (53.4–57.3)	57.1 (54.9–59.4)	37.3 (29.1–46.3)	70.3 (67.4–73.1)	67.9 (54.2–79)	68 (67.1–68.9)
Should health professionals have specific knowledge on cessation techniques?	96.8 (96–97.4)	98.2 (97.5–98.7)	98.3 (93.5–99.6)	94.6 (93.2–95.8)	96.4 (86.2–99.1)	94 (93.5–94.5)
During your (medical, dental, nursing, or pharmacy) school training, were you taught in any of your classes about the dangers of smoking?	84.2 (79.9–87.8)	88.5 (81.2–93.5)	96.6 (90.8–99.0)	79.3 (74.2–84.0)	71.4 (57.5–83.2)	82.0 (73.1–89.0)
During your (medical, dental, nursing, or pharmacy) school training, did you learn that it is important to record tobacco use history as part of a patient’s general medical history?	75.8 (71.0–80.1)	99.1 (95.4–100.0)	57.6 (47.7–67.0)	77.7 (72.2–82.2)	51.9 (37.1–65.6)	67.0 (56.9–76.1)
During your (medical, dental, nursing, or pharmacy) school training, have you ever received any formal training in smoking cessation approaches to use with patients?	13.5 (10.2–17.5)	19.6 (12.7–27.7)	11.9 (6.5–19.6)	22.5 (17.8–27.8)	10.7 (4.2–22.9)	25.0 (16.9–34.7)
During your (medical, dental, nursing, or pharmacy) school training, did you learn that it is important to provide educational materials to support smoking cessation to patients who want to quit smoking?	31.7 (27.0–36.8)	34.8 (26.1–43.9)	43.1 (33.6–53.1)	43.7 (37.9–49.8)	22.2 (11.3–35.5)	46.0 (36.0–56.3)
Have you ever heard of using nicotine replacement therapies in tobacco cessation programs (such as nicotine patch or gum)?	85.5 (81.4–88.9)	83.0 (75.2–89.4)	93.2 (87.1–97.4)	77.5 (72.3–82.4)	82.1 (70.0–91.9)	88.0 (80.0–93.6)
Have you ever heard of using antidepressants in tobacco cessation programs (such as Bupropion or Zyban)?	24.2 (19.9–29.0)	21.4 (14.1–29.6)	23.7 (16.3–33.1)	25.2 (20.3–30.7)	33.3 (20.7–47.9)	17.2 (10.3–26.1)

## 4. Discussion

This study revealed a high percentage of health profession students that were smokers in Greece. The results of the Greek GHPSS show that health visitor students had the highest prevalence (46.4%) of current cigarette smoking while the lowest was among pharmacy students (27.1%). Use of tobacco products was highest among health visitor students (46.4%). Tobacco use endangers the health of health professions students and negatively influences the future health professions workforce to deliver effective anti-tobacco counseling when they start seeing patients [10]. The tobacco control community should target tobacco users among health professions students to overcome this situation. Educational institutions offering health professions courses should help their students quit using tobacco by providing encouragement, information and any other assistance necessary to support them in their efforts to quit smoking. 

The proportion of health professions students in Greece that reported they were exposed to SHS in public places for an entire week was significantly high, ranging from 28.3% among nursing students to 53.3% among health visitors. The findings regarding school smoking regulations were particularly important since many students were not even aware of a smoking ban in their schools. The rates of nutrition and medical students who indicated “I don’t know” to the relevant questions were 42.9% and 40.4% respectively. Further results of the study indicated that the majority of those respondents who were aware of anti-smoking regulations in their schools reported relatively low enforcement of the regulations in place. In particular, 52.2% of dental students stated that smoking is not allowed neither inside school buildings nor inside clinics and 34.9% indicated lack of enforcement of the relevant regulations. 

It is worth noting that at the time of the study national smoke free measures had been adopted through relevant legislation in December, 2008 while the survey was completed approximately at the same time the anti-smoking law came into force (July, 2009). Since then additional measures have been introduced as part of a comprehensive smoke-free legislation, which banned smoking in all closed spaces (with few exceptions) and was voted for in August, 2010 and came into effect soon after (September, 2010). Considering that four schools participated in the GHPSS study during the period October 2010-May 2011, it would be interesting to compare these new results with those of this GHPSS study and draw conclusions on the level of awareness of the new legislation, among the respondents and their opinions regarding its enforcement in their work and study environments (*i.e.*, before and after introduction of the recent comprehensive smoke free policy). However, a possible limitation of our study should be mentioned for interpreting our results: the overall response rate was low particularly for medical, dental and pharmacy schools. 

Educational institutions training health professions students should be encouraged to provide smoke free work environments. A smoke free work environment has been shown to improve air quality, reduce health problems associated with exposure to tobacco smoke, support and encourage cessation attempts among smokers trying to quit, and receive high levels of public support from people who spend time in the area [13]. Furthermore, the creation of smoke free areas by health education institutions sends a clear message to educators, students, patients, and clinicians about the negative impact of tobacco use [14].

Results of the international GHPSS study conducted among health professions students revealed that 20% were current smokers and the majority believed that they should receive formal training on counseling their patients to quit tobacco. However, less than 40% of the students reported they have received such training during their undergraduate studies [12,15,16,17]. Another, recent GHPSS study conducted in Lebanon among health professions students showed that prevalence of smoking among nursing students was 26.9%, and that nine in 10 health professions students believed that they should receive training on smoking cessation techniques, percentages similar to those found within the context of our study [18]. In the same study the percentage of nursing students that reported that they had received training on tobacco related issues during their undergraduate studies reached almost 50% [18]. 

It is critical to examine the perceptions towards tobacco control by health students as they form their professional roles and develop their basic practices while at university [16,17]. Health professionals in Greece have a special role in the health care system and the main responsibility to perform health promotional activities. It is therefore essential to provide them with the required education, knowledge, skills and capacity in order to familiriase their patients with the concept of health promotion and raise awareness not only about the dangers of tobacco use but also about the damaging effects of SHS. In Greece there is a lack in all health professions departments of an educational module on tobacco control. Knowledge on the basic community-based and clinical science related to tobacco use, attitudes and behaviors are basic skills that should be core graduation requirements for health professions students. In the USA it has been recommended to include tobacco cessation counseling techniques in the training program aimed at nurses and that their contribution in this context will be of critical importance in the decrease of tobacco use in the country [16,17]. 

Health professions students should be trained to provide effective, accurate, and accessible advice to patients on all aspects of health. The Greek GHPSS results varied greatly in this part of the questionnaire. 70.3% of nursing students and 37.3% of pharmacy students recognized that they are role models in society. The vast majority of the respondents thought they should receive training on counseling and treating patients to quit using tobacco. However, only 13.5% of medical students, 19.6% of dental students, 11.9% of pharmacy students, 22.4% of nursing students, 10.7% of health visitor students and 25.0% of nutrition students have attended some seminars on this topic. 

There is a need for the development of a specific curriculum to teach students on how to assist smokers to quit and how to counsel non-smoking adolescents so as to prevent them from starting to smoke. These goals have been achieved in some countries that have a core competency and learning curriculum for tobacco education in medical schools, nursing schools could follow. Curricula should include a course or supplements to existing courses specifically relevant to tobacco issues. If administrators are resistant to making changes in the core curricula, schools should be encouraged to incorporate tobacco-related modules within existing courses. 

The majority of evaluation research conducted on tobacco-related curricula has been conducted in high income countries. Relatively little information about the process of teaching health professions students in low and middle-income countries about smoking prevention and cessation is accessible to the international tobacco control community. Peer-reviewed studies in international settings about educational materials and techniques to improve the capacity of health professions students to treat and counsel patients on cessation are necessary to focus resources on effective and efficient strategies to reduce the prevalence of tobacco use. Efforts should be made to assess and share the content of tobacco control components within the formal training curricula and continuing education courses for health professions students. 

## 5. Conclusions

The relatively high percentage of health profession students who currently smoke and the alarmingly high percentage of those exposed to SHS on a daily basis indicate lack of concerted efforts for implementation and effective enforcement of the anti tobacco policy measures. Educational institutions, public health organizations and education officials should discourage tobacco use among health professions students by raising awareness and educating them on the dangers associated with tobacco use and exposure to SHS. All relevant institutions and organizations should work together to design and implement training programs for health professions students that will enable them to develop the necessary skills and capacity to provide effective tobacco cessation support and counseling techniques to their future patients. 

## Competing Interests

The authors declare no conflict of interest.

## Supplementary Files

Supplementary File 1: PDF-Document (PDF, 217 KB)
