# Correction: Barbouni, A., *et al*. Tobacco Use, Exposure to Secondhand Smoke, and Cessation Counseling Among Health Professions Students: Greek Data from the Global Health Professions Student Survey (GHPSS). *Int. J. Environ. Res. Public Health* 2012*, 9, *331-342

**DOI:** 10.3390/ijerph9093317

**Published:** 2012-09-10

**Authors:** Anastasia Barbouni, Christos Hadjichristodoulou, Kyriakoula Merakou, Eleni Antoniadou, Kallirrhoe Kourea, Evangelia Miloni, Charles W. Warren, George Rachiotis, Jenny Kremastinou

**Affiliations:** 1 Department of Public Health, National School of Public Health, Alexandras Av. 196, Athens 11521, Greece; Email: kmerakou@esdy.edu.gr (K.M.); eantoniadou@gmail.com (E.A.); publichealth@esdy.edu.gr (K.K.); m-kelly@live.com (E.M.); jkremastinou@esdy.edu.gr (J.K.); 2 Department of Hygiene and Epidemiology, Faculty of Medicine, University of Thessaly, Papakyriazi 22, Larissa 41222, Greece; Email: xhatzi@med.uth.gr (C.H.); grach@med.uth.gr (G.R.); 3 Office of Smoking and Health, National Center for Chronic Disease Prevention and Health Promotion, Centers for Disease Control and Prevention, 1600 Clifton Rd. Atlanta, GA 30333, USA; Email: wcw1@cdc.gov

The authors wish to make the following correction to this paper [1]: The author name “George Rahiotis” should be “George Rachiotis”. 

## References

[1] Barbouni A., Hadjichristodoulou C., Merakou K., Antoniadou E., Kourea K., Miloni E., Warren C.W., Rahiotis G., Kremastinou J. (2012). Tobacco use, exposure to secondhand smoke, and cessation counseling among health professions students: Greek data from the Global Health Profession Student Survey (GHPSS). Int. J. Environ. Res. Public Health.

