# Supplementary material for: Tobacco Use, Exposure to Secondhand Smoke, and Cessation Counseling Among Health Professions Students: Greek Data from the Global Health Professions Student Survey (GHPSS)
Source: Int J Environ Res Public Health. 2012 Jan 19;9(1):331–42. doi: 10.3390/ijerph9010331 (PMC3315079; doi:10.3390/ijerph9010331)
Supplement: Supplementary File 1: — PDF-Document (PDF, 217 KB) [file ijerph-09-00331-s001.pdf]

Int. J. Environ. Res. Public Health **2012**, *9*, 331-342; doi:10.3390/ijerph9010331

OPEN ACCESS

International Journal of
Environmental Research and Public Health
ISSN 1660-4601
www.mdpi.com/journal/ijerph

Article

Tobacco Use, Exposure to Secondhand Smoke, and Cessation Counseling Among Health Professions Students: Greek Data from the Global Health Professions Student Survey (GHPSS)

Anastasia Barbouni [1,*], Christos Hadjichristodoulou [2], Kyriakoula Merakou [1], Eleni Antoniadou [1], Kallirrhoe Kourea [1], Evangelia Miloni [1], Charles W. Warren [3], George Rahiotis [2] and Jenny Kremastinou [1]

[1] Department of Public Health, National School of Public Health, Alexandras Av. 196, 11521, Athens, Greece; E-Mails: kmerakou@esdy.edu.gr (K.M.); eantoniadou@gmail.com (E.A.); publichealth@esdy.edu.gr (K.K.); m-kelly@live.com (E.M.); jkremastinou@esdy.edu.gr (J.K.)

[2] Department of Hygiene and Epidemiology, Faculty of Medicine, University of Thessaly, Papakyriazi 22, 41222, Larissa, Greece; E-Mails: xhatzi@med.uth.gr (C.H.); grach@med.uth.gr (G.R.)

[3] Office of Smoking and Health, National Center for Chronic Disease Prevention and Health Promotion, Centers for Disease Control and Prevention, 1600 Clifton Rd. Atlanta, GA 30333, USA; E-Mail: wcw1@cdc.gov

* Author to whom correspondence should be addressed; E-Mail: abarbouni@esdy.edu.gr; Tel.: +30-21-2010255; Fax: +30-21-06423041.

Received: 13 December 2011; in revised from: 13 January 2012 / Accepted: 16 January 2012 / Published: 19 January 2012

Abstract: We conducted the GHPSS (Global Health Professions Student Survey) to obtain information regarding health profession students' smoking habits and perceptions, exposure to secondhand smoke (SHS) as well as level of knowledge and training on tobacco use and smoking cessation counseling. GHPSS is a survey for third-year students in the following fields: health visitors, dentistry, medicine, nursing and/or pharmacy. The highest tobacco use prevalence rate and exposure to SHS were recorded among health visitor students with 46.4% and 33.3% respectively. The majority of the respondents believed that their profession serves as a role model for their patients. Formal training on cessation counseling ranged between 10.7% for health visitor students to 22.4% for nursing students. The relatively high percentage of health profession students who currently smoke and the alarmingly high percentage of those exposed to SHS indicate lack of concerted efforts for implementation

and effective enforcement of the anti-tobacco policy measures. Despite its significance, formal training on cessation counseling for students is strikingly low. These results indicate the urgent need to train health professional students on tobacco cessation counseling and educate them on the dangers of tobacco use, SHS and the positively influential role they can play to affect their patients' smoking habits.

Keywords: tobacco; smoking; prevalence; students; secondhand smoke; health profession; cessation; survey

---

National School of Public Health
Department: Public Health and Management of Health
Professor: Jenny Kourea-Kremastinou

(GLOBAL HEALTH PROFESSIONS STUDENT SURVEY, GHPSS)

World Health Organization (WHO)
Center for Disease Control (CDC)

Directions for the completion of the questionnaire

- ❑ Read each question carefully before you answer
- ❑ Choose the answer which best describes what you believe is the right answer
- ❑ Choose only **one** answer for each question
- ❑ On your response sheet, find the circle which needs to be filled in for your answer and completely fill it in with the pencil you were given
- ❑ Fill in the circles correctly, like this–⦿
- ❑ If you need to change your answer, do not worry, simply erase your answer completely without leaving any marks
- ❑ Remember that each question only has **one** answer

I. Individual Smoking Behavior

1. Have you ever smoked a cigarette, even just once or twice?
   a. Yes
   b. No
2. How old were you when you smoked for the first time?
   a. I have never smoked a cigarette
   b. Less than 10 years
   c. Ages 11-15
   d. Ages 16-17
   e. Ages 18-19
   f. Ages 20-24
   g. Ages 25-29
   h. 30 and older

3. During the past 30 days (one month), how many days did you smoke cigarettes?
    a. O days
    b. 1 or 2 days
    c. 3 to 5 days
    d. 6 to 9 days
    e. 10 to 19 days
    f. 20 to 29 days
    g. The entire month (all thirty days)
4. During the past year, have you ever smoked around the area of the School?
    a. I have never smoked cigarettes
    b. Yes
    c. No
5. During the past year, have you ever smoked inside the buildings of the School?
    a. I have never smoked cigarettes
    b. Yes
    c. No
6. Have you ever tried chewing tobacco?
    a. Yes
    b. No
7. Have you ever tried bibis?
    a. Yes
    b. No
8. Have you ever tried smoking a cigar?
    a. Yes
    b. No
9. Have you ever tried smoking a pipe?
    a. Yes
    b. No
10. During the past 30 days (one month), how many days did you use chewing tobacco?
    a. O days
    b. 1 or 2 days
    c. 3 to 5 days
    d. 6 to 9 days
    e. 10 to 19 days
    f. 20 to 29 days
    g. The entire month (all thirty days)

11. During the past 30 days (one month), how many days have you used bibis?
    a. O days
    b. 1 or 2 days
    c. 3 to 5 days
    d. 6 to 9 days
    e. 10 to 19 days
    f. 20 to 29 days
    g. The entire month (all thirty days)
12. During the past 30 days (one month), how many days did you use a cigar?
    a. O days
    b. 1 or 2 days
    c. 3 to 5 days
    d. 6 to 9 days
    e. 10 to 19 days
    f. 20 to 29 days
    g. The entire month (all thirty days)
13. During the past 30 days (one month), how many days did you use a pipe?
    a. O days
    b. 1 or 2 days
    c. 3 to 5 days
    d. 6 to 9 days
    e. 10 to 19 days
    f. 20 to 29 days
    g. The entire month (all thirty days)
14. During the past year, have you ever used chewing tobacco around the area of the School?
    a. I have never smoked cigarettes
    b. Yes
    c. No
15. During the past year, have you ever used bibis around the area of the School?
    a. I have never smoked cigarettes
    b. Yes
    c. No
16. During the past year, have you ever used a cigar around the area of the School?
    a. I have never smoked cigarettes
    b. Yes
    c. No
17. During the past year, have you ever used a pipe around the area of the School?
    a. I have never smoked cigarettes
    b. Yes
    c. No

18. During the past year, have you ever used chewing tobacco inside the buildings of the School?
    a. I have never smoked cigarettes
    b. Yes
    c. No
19. During the past year, have you ever used bibis inside the buildings of the School?
    a. I have never smoked cigarettes
    b. Yes
    c. No
20. During the past year, have you ever used a cigar inside the buildings of the School?
    a. I have never smoked cigarettes
    b. Yes
    c. No
21. During the past year, have you ever used a pipe inside the buildings of the School?
    a. I have never smoked cigarettes
    b. Yes
    c. No

II. Exposure to cigarette smoke in the environment

22. During the past 7 days at the place where you live, how many days did other individuals smoke in front of you?
    a. 0 days
    b. 1 to 2 days
    c. 3 to 4 days
    d. 5 to 6 days
    e. All 7 days
23. During the past 7 days in public areas, how many days did other individuals smoke in front of you?
    a. 0 days
    b. 1 to 2 days
    c. 3 to 4 days
    d. 5 to 6 days
    e. All 7 days
24. Does your School have specific regulations which prohibit smoking inside the building and clinics?
    a. Yes, only for the buildings of the School
    b. Yes, only for the clinics
    c. Yes, for both the buildings and the clinics
    d. There are no specific regulations
    e. I do not know

25. Is the no-smoking regulation followed at your School?
    a. Yes, the regulation is followed
    b. No, the regulation is not followed
    c. There is no anti-smoking regulation at the School

III. Opinions / Lifestyle

26. Should selling cigarettes to minors (individuals less than 18 years of age) be prohibited?
    a. Yes
    b. No
27. Should all forms of smoking related advertisement be prohibited?
    a. Yes
    b. No
28. Should smoking be prohibited in restaurants?
    a. Yes
    b. No
29. Should smoking be prohibited at cafes, discos, nightclubs, bars, etc?
    a. Yes
    b. No
30. Should smoking be prohibited in all indoor areas?
    a. Yes
    b. No
31. Should health professionals have specific knowledge relating to smoking cessation techniques?
    a. Yes
    b. No
32. Do you believe that health professionals are "role models" for their patients and the larger public?
    a. Yes
    b. No
33. Should health professionals repeatedly advise their patients to quit smoking?
    a. Yes
    b. No
34. Should health professionals repeatedly advise their patients that use other types of smoke to stop their use?
    a. Yes
    b. No
35. Do you believe that health professionals have the ability and the position as a role model to provide advice and information to their patients in terms of smoking cessation?
    a. Yes
    b. No

36. Does the possibility that a patient may quit smoking increase if a health professional advises him/her?
    a. Yes
    b. No

IV. Behavior / Cessation

37. How long after you wake up in the morning to you smoke your first cigarette?
    a. I have never smoked cigarettes
    b. During this period I do not smoke cigarettes
    c. 10 minutes or less
    d. 10-30 minutes
    e. 31-60 minutes
    f. After 60 minutes
38. During this time period do you want to quit smoking?
    a. Yes
    b. No
    c. I have never smoke cigarettes
39. During the past year, have you tried to quite smoking?
    a. I have never smoked cigarettes
    b. I have not smoked this past year
    c. Yes
    d. No
40. How long has it been since you quite smoking?
    a. I have never smoked cigarettes
    b. I have not quite smoking
    c. Less than a month
    d. 1-5 months
    e. 6-11 months
    f. One year
    g. 2 years
    h. 3 or more years
41. Have you ever accepted help or advice to quit smoking?
    a. I have never smoked cigarettes
    b. Yes
    c. No
42. Do you want to stop using chewing tobacco?
    a. I have never used chewing tobacco
    b. I do not use chewing tobacco
    c. Yes
    d. No

43. Do you want to stop using bibis?
    a. I have never used bibis
    b. I do not use bibis
    c. Yes
    d. No
44. Do you want to stop using cigars?
    a. I have never used a cigar
    b. I do not use cigars
    c. Yes
    d. No
45. Do you want to stop using a pipe?
    a. I have never used a pipe
    b. I do not use a pipe
    c. Yes
    d. No
46. Do you believe that health professionals successfully advise their patients to quit smoking cigarettes?
    a. Yes
    b. No
47. Do you believe that health professionals successfully advise their patients who smoke other forms of smoke products (i.e.smoke, bibis, cigarettes, cigars, and pipes) to quit?
    a. Yes
    b. No

V. <u>School Program / Education</u>

48. During your studies, did any class teach you about the risks that smoking presents?
    a. Yes
    b. No
49. During your studies, did any of your classroom discussions include the reasons why people choose to smoke?
    a. Yes
    b. No
50. During your studies, have you learned that it is very important to document the smoking history of a patient as a part of their general health history?
    a. Yes
    b. No
51. During your studies, have you ever received any training regarding techniques that a patient can use to quit smoking?
    a. Yes
    b. No

52. During your studies, have you learned that it is very important to provide patients who want to quit smoking with various material and information regarding smoking?
    a. Yes
    b. No
53. Have you ever heard of nicotine replacement therapy in cessation programs (for example, nicotine patches or gum with nicotine)?
    a. Yes
    b. No
54. Have you ever heard of anti-depressant medication in cessation programs (like buproprion, Zyban)?
    a. Yes
    b. No

VI. Personal Information

55. How old are you?
    a. 14 or younger
    b. 15-18 years
    c. 19-24 years
    d. 25-29 years
    e. 30 or younger
56. What gender are you?
    a. Woman
    b. Man
57. What is your current year of study?
    a. First
    b. Second
    c. Third
    d. Fourth
    e. Fifth
    f. Sixth
    g. Seventh

© 2012 by the authors; licensee MDPI, Basel, Switzerland. This article is an open access article distributed under the terms and conditions of the Creative Commons Attribution license (http://creativecommons.org/licenses/by/3.0/).
